# Expression analysis of candidate genes regulating successional tooth formation in the human embryo

**DOI:** 10.3389/fphys.2014.00445

**Published:** 2014-11-21

**Authors:** Ryan Olley, Guilherme M. Xavier, Maisa Seppala, Ana A. Volponi, Fin Geoghegan, Paul T. Sharpe, Martyn T. Cobourne

**Affiliations:** ^1^Department of Conservative Dentistry, Dental Institute, King's College LondonLondon, UK; ^2^Department of Craniofacial Development and Stem Cell Biology, Dental Institute, King's College LondonLondon, UK; ^3^Department of Orthodontics, Dental Institute, King's College LondonLondon, UK; ^4^Department of Craniofacial Development and Stem Cell Biology, King's College LondonLondon, UK

**Keywords:** human tooth development, three-dimensional reconstruction, *SPROUTY2*, *GAS1*, *RUNX2*, primary dental lamina, successional dental lamina, gene expression

## Abstract

Human dental development is characterized by formation of primary teeth, which are subsequently replaced by the secondary dentition. The secondary dentition consists of incisors, canines, and premolars, which are derived from the successional dental lamina of the corresponding primary tooth germs; and molar teeth, which develop as a continuation of the dental lamina. Currently, very little is known about the molecular regulation of human successional tooth formation. Here, we have investigated expression of three candidate regulators for human successional tooth formation; the Fibroblast Growth Factor-antagonist *SPROUTY2*, the Hedgehog co-receptor *GAS1* and the RUNT-related transcription factor *RUNX2*. At around 8 weeks of development, only *SPROUTY2* showed strong expression in both epithelium and mesenchyme of the early bud. During the cap stage between 12–14 weeks, *SPROUTY2* predominated in the dental papilla and inner enamel epithelium of the developing tooth. No specific expression was seen in the successional dental lamina. *GAS1* was expressed in dental papilla and follicle, and associated with mesenchyme adjacent to the primary dental lamina during the late cap stage. In addition, *GAS1* was identifiable in mesenchyme adjacent to the successional lamina, particularly in the developing primary first molar. For *RUNX2*, expression predominated in the dental papilla and follicle. Localized expression was seen in mesenchyme adjacent to the primary dental lamina at the late cap stage; but surprisingly, not in the early successional lamina at these stages. These findings confirm that *SPROUTY2*, *GAS1*, and *RUNX2* are all expressed during early human tooth development. The domains of *GAS1* and *RUNX2* are consistent with a role influencing function of the primary dental lamina but only *GAS1* transcripts were identifiable in the successional lamina at these early stages of development.

## Introduction

Vertebrates demonstrate wide variation in the functional requirements of their masticatory systems and this is reflected in the anatomic variation within their dentitions. One area of significant diversification is the capacity to regenerate teeth, with some vertebrate species able to replace teeth throughout life, whilst others produce only a single dentition over their lifetime (Tucker and Fraser, 2014).

Amongst the mammals, tooth replacement is rarely carried out more than once, which is thought to reflect the increased complexity of tooth shape and occlusion that is seen in these animals (Jarvinen et al., 2009b). The mammalian dentition is classically heterodont, with incisor, canine, and molariform teeth present in both the primary and secondary dentitions. In many mammals, including humans and other primates, the transition from a primary to permanent dentition is achieved through the generation of successional incisor, canine and premolar teeth, which are derived from a successional dental lamina that forms on the lingual side of the corresponding primary tooth germ (Berkovitz et al., 2009; Ten Cate, 2014). In contrast, the secondary molar dentition is accessional, the first molar initiating from a posterior extension of the primary dental lamina and subsequent molars budding off through a process termed serial addition (Juuri et al., 2013).

The development of individual teeth within the primary and secondary dentitions is characterized by a series of reiterative molecular interactions that take place between odontogenic epithelium and neural crest-derived ectomesenchyme within the early jaw primordia (Jernvall and Thesleff, 2000, 2012; Tucker and Sharpe, 2004). The mouse has been used very successfully to identify many of the molecular signaling interactions that are required to generate a primary tooth. However, the murine dentition is monophyodont, with mice only generating one set of primary teeth during their lifetime. Moreover, this dentition is highly reduced, with only incisor and molar teeth present, separated by an edentulous diastema (Lesot et al., 2014; Peterkova et al., 2014). As a consequence, the mouse is a less informative model of tooth replacement; although, recently a number of mouse mutants have been described with supernumerary premolar teeth that form within the diastema region (Klein et al., 2006; Ohazama et al., 2009; Ahn et al., 2010). Current evidence suggests that these may represent vestigial teeth, arising from early premolar tooth germs that are programmed to regress in the wild type mouse (Peterkova et al., 2014). Analysis of these mutants has demonstrated the presence of complex interactions between WNT, FGF, and Hedgehog signaling pathways that ultimately dictate whether these teeth arrest or proceed beyond the bud stage to form a definitive tooth (Klein et al., 2006; Ohazama et al., 2009; Ahn et al., 2010).

In order to circumvent the problems associated with the mouse as a model of successional tooth replacement, researchers have utilized a number of alternative mammalian models that do develop a secondary dentition, including the Shrew and Ferret. However, the Shrew primary dentition is essentially transient (Yamanaka et al., 2010) and non-functional and whilst the Ferret does generate two functional dentitions (Jarvinen et al., 2009a; Jussila et al., 2014) it is a less accessible animal model and neither species offers the same opportunities for genetic manipulation currently available in the mouse. A further potential model for investigating tooth replacement is provided by reptiles, where replacement teeth also arise from a successional dental lamina. Reptilian embryos are accessible during development and this has been exploited in a variety of species to investigate successional tooth replacement (Buchtova et al., 2008; Handrigan and Richman, 2010a,b). Currently, little is known about the mechanisms that govern human successional tooth formation.

Here, we have investigated the expression of three candidate genes potentially implicated in the regulation of successional tooth formation in human tooth development. In recent years, a number of mouse mutants have been described with supernumerary premolar teeth situated in front of the first molars, which occur with varying levels of penetrance and may represent the re-emergence of a vestigial dentition (Klein et al., 2006; Ohazama et al., 2009; Ahn et al., 2010). Interestingly, the mutated genes are known to regulate four of the major signaling pathways that are active during murine tooth development [WNT, Hedgehog, Fibroblast Growth Factor (FGF) and Bone Morphogenetic Protein (BMP)] and there is evidence that thresholds of signal activity ultimately dictate whether supernumerary tooth formation takes place or not (Ahn et al., 2010; Charles et al., 2011). In particular, negative regulation of WNT and BMP signaling through the induction of Sonic hedgehog and Sostdc1 and as a consequence, tempering of FGF signal levels can dictate whether these teeth form or not (Klein et al., 2006; Ohazama et al., 2009; Ahn et al., 2010). Within these pathways, *Sprouty2* encodes an FGF signaling antagonist (Klein et al., 2006) and *Gas1* encodes a GPI-linked membrane glycoprotein, which acts as a co-receptor in the Hedgehog signaling pathway (Seppala et al., 2007, 2014) and both of these mouse mutants have supernumerary teeth with high penetrance. In humans, there are few candidate genes for supernumerary tooth formation; however, *RUNX2* encodes a RUNT-related transcription factor, which is mutated in Cleidocranial Dysplasia [**#**119600], a human skeletal dysplasia characterized by the presence of multiple supplemental supernumerary teeth affecting the secondary dentition (Komori et al., 1997; Lee et al., 1997; Mundlos et al., 1997). We have investigated expression of these genes during early human tooth development using *in situ* hybridization. We find that all three genes are expressed in developing primary teeth. *SPROUTY2* predominates in the dental papilla and internal enamel epithelium at the cap stage. *GAS1* is expressed in both the dental papilla and follicle, and is upregulated in mesenchyme adjacent to the primary and successional dental laminas. *RUNX2* was expressed in mesenchyme adjacent to the primary dental lamina and in both the dental papilla and follicle. These findings demonstrate that all three of these genes are expressed during human tooth development with the expression domains of *GAS1* and *RUNX2* consistent with a role influencing formation of the secondary dentition.

## Materials and methods

### Embryo collection

Human embryos were obtained at a variety of stages of gestation (approximately 8–14 weeks post-fertilization) from the Human Developmental Biology Resource Birth Defects Research Centre at the Institute of Child Health, University College London. All embryos were derived from elective termination of pregnancy. The general ethical approval is held by UCL Institute of Child Health; King's College London has a subscription to obtain embryos from this center. Embryos were stored in phosphate buffered saline and delivered immediately following retrieval via courier.

### Histological analysis

For histological analysis, embryos were fixed in 4% paraformaldehyde (PFA) at 4°C and decalcified in 10% EDTA (pH 7.4) for 8–12 weeks at 4°C (depending upon stage). Following this, they were dehydrated through a graded ethanol series, embedded in paraffin wax, sectioned at 7 μm and mounted on slides prior to either staining with haematoxylin and eosin or preparation for section *in situ* hybridization.

### Three-dimensional reconstruction

Images from consecutive haematoxylin and eosin-stained histological sections were used to create a three-dimensional reconstruction of the developing primary teeth (enamel organs) and their successional lamina using DeltaViewer 2.1 3D imaging software. DeltaViewer reads a sequence of cross-sectional images of an object and uses these to computationally reconstruct the object. Images were imported from Adobe Photoshop version 8 into DeltaViewer 2.1. Single consecutive images were stacked and aligned using the boundary of the oral epithelium and dental lamina, and the extension of the midline of the tooth germ as alignment points. The painted white areas on each consecutive image were selected and slices of the aligned stacks were saved as files. The software then created a three-dimensional reconstruction of the tooth germ, its successional lamina and the overlying oral epithelium. The reconstructed surface was then smoothed, visualized in three-dimensions and saved as a QuickTime 7.7.5 (Apple Corp, USA) movie files. Static images of the three-dimensional reconstructions were then taken.

### *In situ* hybridisation

Radioactive section *in situ* hybridisation was carried out as previously described (Wilkinson, 1992). Human cDNA IMAGE clones for *SPROUTY2*, *GAS1*, and *RUNX2* were obtained from Source Bioscience. Light and dark-field images of sections were photographed using a Zeiss Axioscop microscope and merged in Adobe Photoshop CS.

## Results

**Figure 1:**
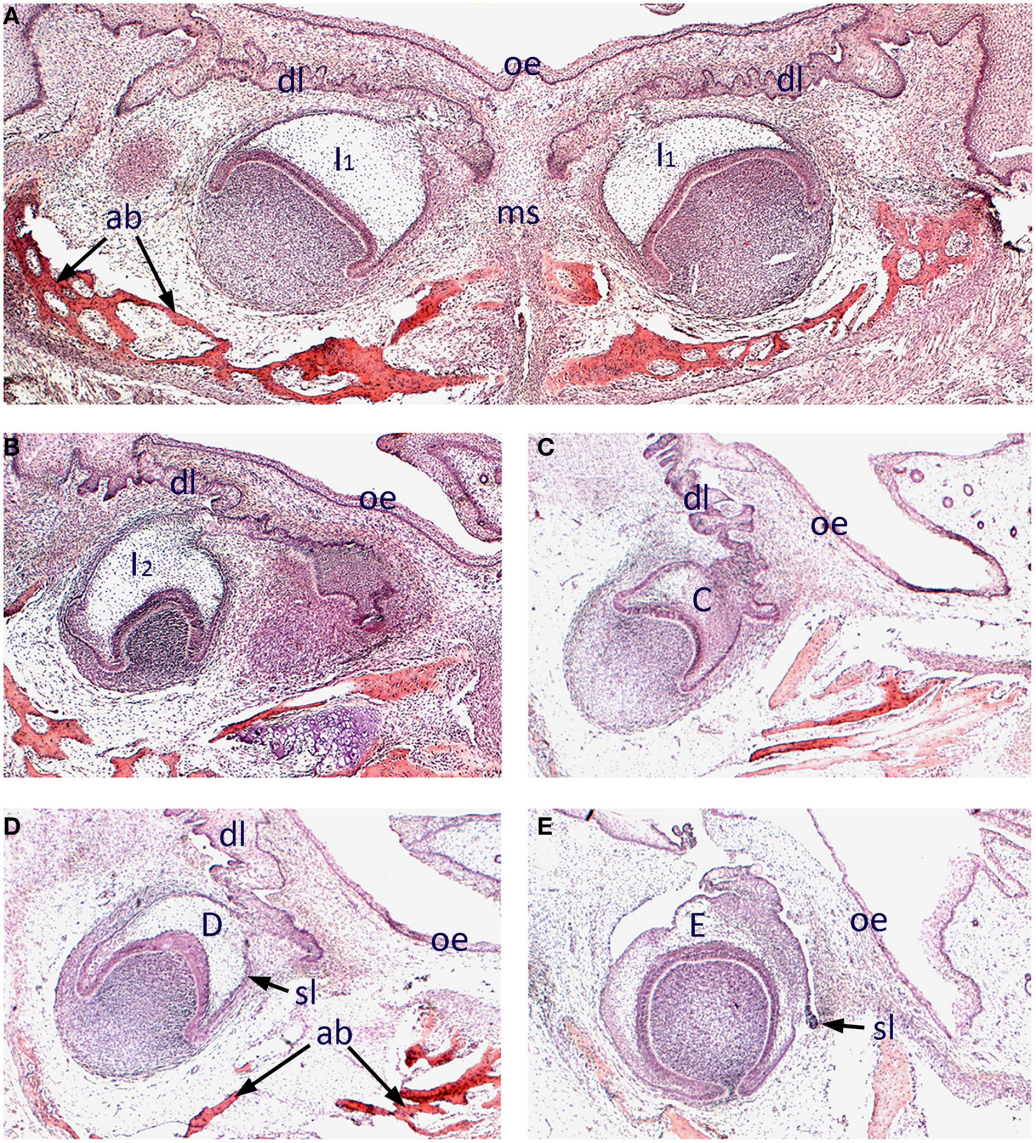
**Histology of human tooth development**. Frontal sections through the mandible of a human embryo at approximately 12 weeks of development (stained with Hematoxilin and Eosin). **(A)** Primary central incisor tooth germs in the developing mandibular symphysis; **(B)** Primary lateral incisor tooth germ; **(C)** Primary canine tooth germ; **(D)** Primary first molar tooth germ; **(E)** Primary second molar tooth germ. [ab, alveolar bone; C, primary canine tooth germ; D, primary first molar tooth germ; dl, dental lamina; E, primary second molar tooth germ; I_1_, primary central incisor tooth germ I_2_, primary lateral incisor tooth germ; ms, mandibular symphysis; oe, oral epithelium; sl, successional lamina].

We began by surveying the morphology of early odontogenesis in the human embryo using standard histology and three-dimensional reconstruction. The developing mandibular dentition was investigated at 12 weeks of development using frontal sections. At this stage, the primary central incisors are situated bilaterally in the midline of the early mandible and at the late cap stage of development. These teeth retained a clear attachment to the oral epithelium through the (primary) dental lamina and were closely associated with developing intramembranous bone of the mandibular symphysis (Figure 1A). Further posteriorly, the mandibular primary lateral incisor (Figure 1B), primary canine (Figure 1C) and primary first and second molar (Figures 1D,E) tooth germs were present, appearing further posteriorly in each respective quadrant of the mandible. These teeth were all at the cap stage of development, with the early successional dental laminae associated with the permanent tooth germs visible (Figures 1D,E).

**Figure 2:**
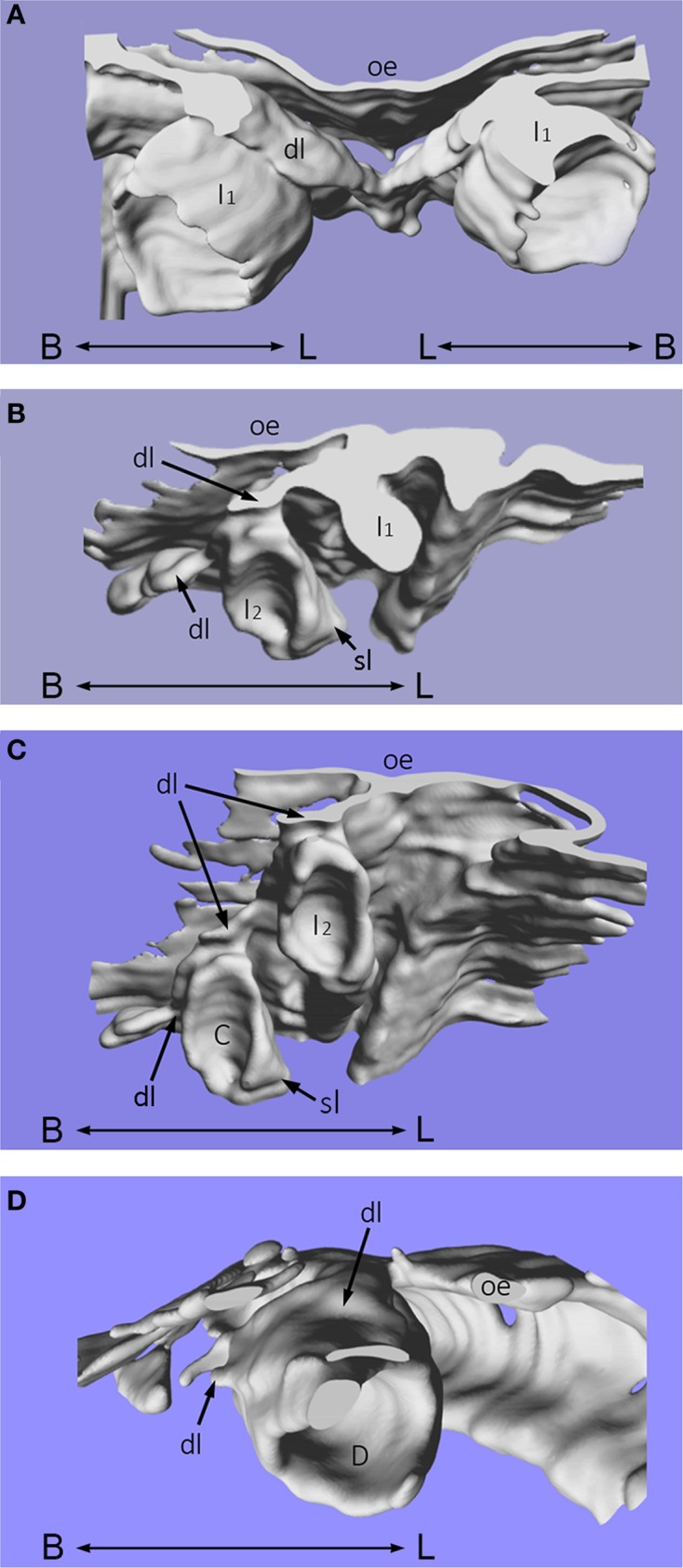
**Three-dimensional reconstruction of human tooth development**. Three-dimensional reconstruction of human tooth development in the mandible at approximately 12 weeks of gestation during the cap stage. **(A)** Primary central incisor tooth germs; **(B)** Primary lateral (and central) incisor tooth germs; **(C)** Primary lateral incisor and canine tooth germs; **(D)** Primary first molar tooth germ. [C, primary canine tooth germ; D, primary first molar tooth germ; dl, dental lamina; I_1_, primary central incisor tooth germ; I_2_, primary lateral incisor tooth germ; oe, oral epithelium; sl, successional lamina; B–L indicates buccal to lingual].

Using computer imaging, it was also possible to reconstruct these histological sections into three-dimensions to visualize the developing enamel organs of the mandibular primary incisors, canine and first molar teeth in more detail (Figures 2A–D). The dental lamina is continuous with the oral epithelium and lies superior to the primary tooth germs. The successional lamina is a lingually-positioned epithelial band, with projections situated at the sites that correspond to each primary tooth germ (Figures 2B,C). From the primary central and lateral incisor enamel organs, the successional lamina is depicted as a lingual projection of epithelium that will later form the mandibular secondary first and second incisor tooth germs, respectively. These successional laminae are positioned near the middle of the primary tooth germs and not deviated either mesially or distally (Figure 2B). Whilst the primary central and lateral incisor tooth germs are in close proximity, the primary canine is deeper in the jaw, positioned widely distal and inferior to the primary lateral incisor enamel organ (Figure 2C). The successional lamina is positioned on the mid-lingual aspect of the primary canine as a projection of epithelium that will go on to form the permanent canine tooth. For the primary first molar tooth germ, the successional dental lamina is situated mesial to the middle part of the primary first molar on the lingual aspect of the tooth germ (Figure 2D). The oral epithelium lies superior to the primary tooth germ and the dental lamina is continuous with it.

### Expression of SPRY2, GAS1, and RUNX2 during human tooth development

**Figure 3:**
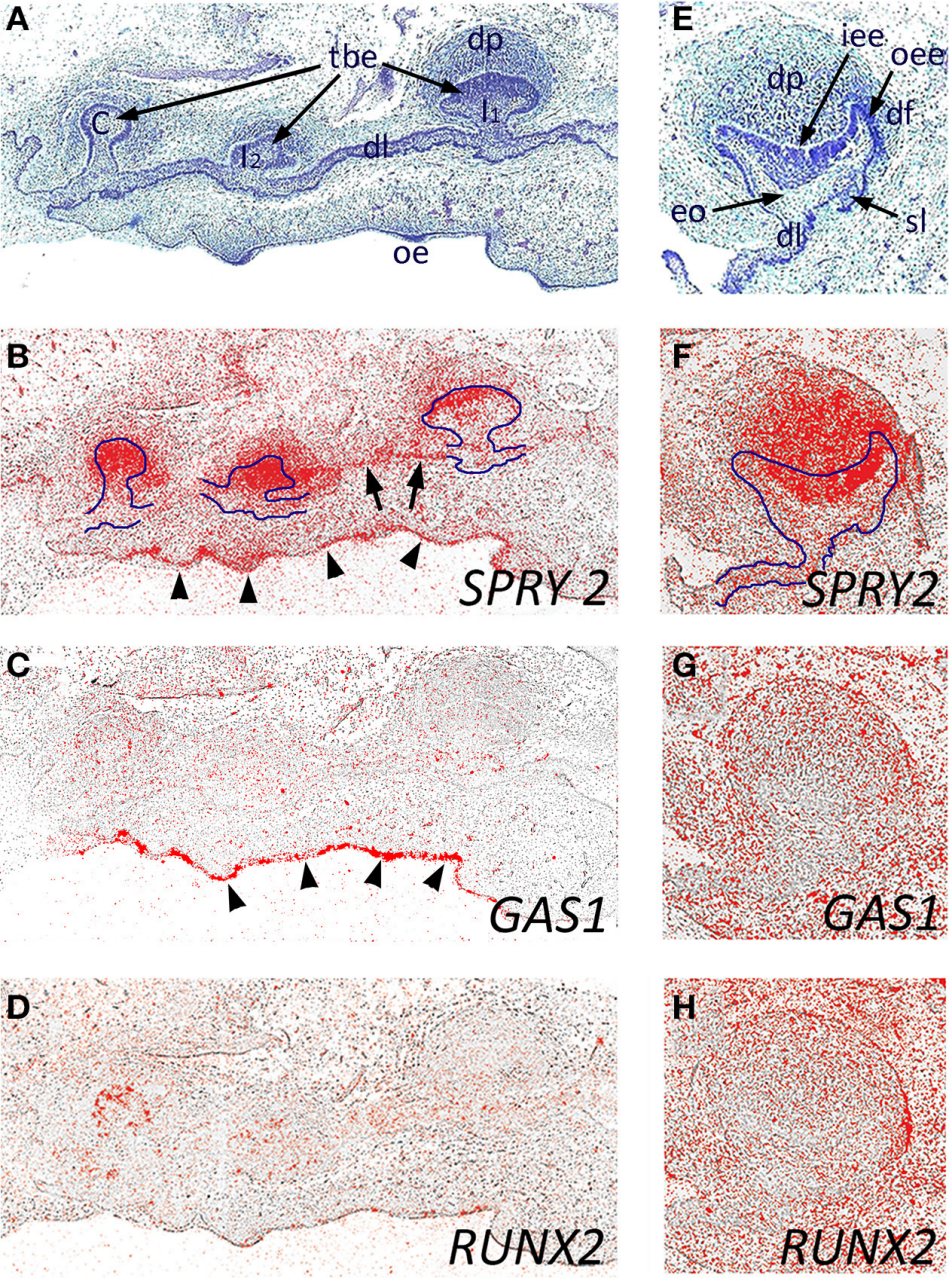
**Candidate gene expression in the developing maxillary incisor and canine dentition of the human embryo**. Frontal sections through the developing primary maxillary incisor and canine region at around 8 weeks of development **(A–D)** and through the primary canine tooth germ at 12 weeks of development **(E–H)** during the early bud and early cap stages, respectively. **(A,E)** Hematoxilin and Eosin; **(B,F)** *SPRY2*; **(C,G)** *GAS1*; **(D,H)** *RUNX2 in situ* hybridization. The early tooth buds and enamel organ are outlined (in **B** and **F**, respectively) to illustrate domains of *SPRY2* expression in both epithelial and mesenchymal structures within the tooth germs. *SPRY2* expression in the dental lamina is arrowed in **(B)**; whilst *SPRY2* and *GAS1* expression in the oral epithelium is defined by arrowheads (**B** and **C**, respectively). [tbe, tooth bud epithelium; C, primary canine tooth germ; dl, dental lamina; dp, dental papilla; df, dental follicle; eo, enamel organ; iee, inner enamel epithelium; oee, outer enamel epithelium; I_1_, primary central incisor tooth germ I_2_, primary lateral incisor tooth germ; oe, oral epithelium; sl, successional lamina].

We next investigated the expression of *SPRY2*, *GAS1*, and *RUNX2* during early human odontogenesis. At around 8 weeks of development, the maxillary primary incisors and canine are at the bud stage (Figures 3A–D), where there was strong expression of *SPRY2* in the outer regions of the tooth bud epithelium and within the condensing mesenchyme of the dental papilla. In addition, weaker expression was also seen in the dental lamina connecting the tooth germs (Figure 3B, arrowed). There was also strong expression of *SPRY2* in the oral epithelium (Figure 3B, arrowheads). In contrast, the expression of *GAS1* was only at background levels in association with the developing tooth germs at this stage, although stronger localized expression was identifiable in the oral epithelium (Figure 3C, arrowheads). *RUNX2* expression was similar to *GAS1* in these tooth germs at the bud stage, but was not present in the oral epithelium (Figure 3D).

We further investigated the expression of these genes at the early cap stage of development in the maxillary primary canine tooth germ at 12 weeks of development (Figures 3E–H). At this stage, *SPRY2* continued to be strongly expressed in the dental papilla and internal enamel epithelium, with weaker expression in the dental lamina and external enamel epithelium (Figure 3F). Both *GAS1* and *RUNX2* expression remained low at this stage, confined to peripheral regions of the tooth follicle (Figures 3G,H). Although the early successional lamina associated with the permanent maxillary canine was identifiable, there was no specific expression of any of these candidate genes at this stage.

**Figure 4:**
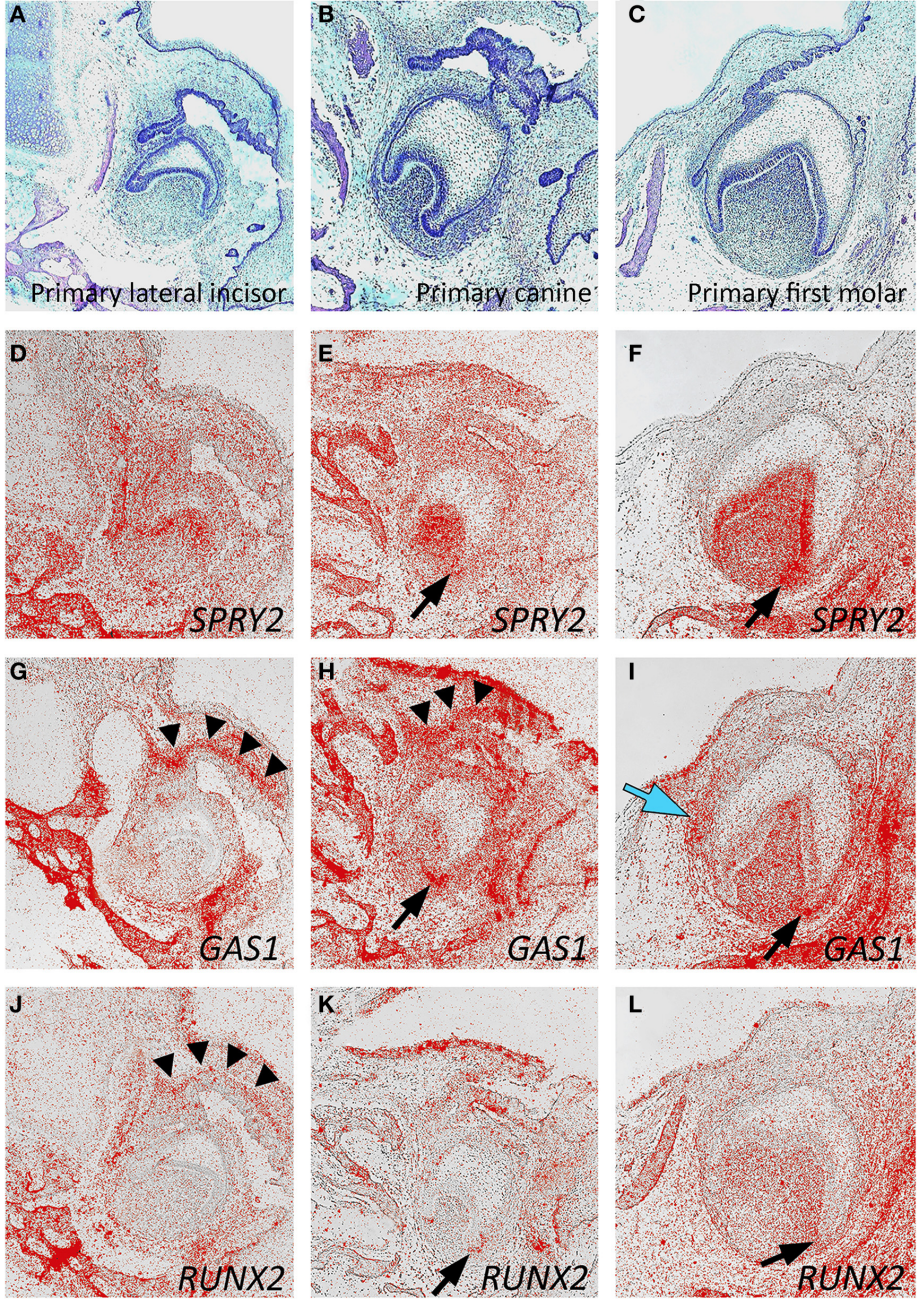
**Candidate gene expression in the developing mandibular dentition of the human embryo**. Frontal sections through the developing primary lateral incisor **(A,D,G,J)**, canine **(B,E,H,K)**, and first molar **(C,F,I,L)** at approximately 14 weeks of development during the late cap stage. **(A–C)** Hematoxilin and Eosin; **(D–F)** *SPRY2*; **(G–I)** *GAS1*; **(J–L)** *RUNX2 in situ* hybridization [black arrowheads show gene expression in the primary dental lamina; black arrows show gene upregulation in the buccal cervical loop; blue arrow shows gene upregulation in the successional lamina].

At around 14 weeks of development, the expression of *SPRY2*, *GAS1*, and *RUNX2* was mapped in the developing mandibular primary lateral incisor, canine, and first molar tooth germs, which had reached the early bell stage of development (Figures 4A–L). In general, the expression domains of these three genes were consistent between different tooth germs, although some subtle differences were seen. For *SPRY2*, expression predominated in the dental papilla and inner enamel epithelium with lower levels in the dental follicle. Expression was also seen in the early alveolar bone of the mandible. No specific expression was seen in the region of the successional dental lamina, which was clearly discernible in the first molar tooth germ (Figures 4D–F). *GAS1* was strongly expressed in mesenchyme directly associated with the dental lamina of the lateral incisor and canine tooth germs. In the first molar, *GAS1* was also upregulated in mesenchyme adjacent to the successional lamina, although not along its entire length. *GAS1* was also expressed in the mesenchyme of the dental papilla and follicle with some increased intensity at the cervical loop and was also seen in the developing alveolar bone (Figures 4G–I). For *RUNX2*, expression remained relatively low in the tooth germs, with the highest activity predominating in the mesenchymal components, including the dental papilla and follicle. No localized or specific expression of *RUNX2* was seen in the region of the successional lamina, although localized expression was seen in mesenchyme adjacent to the primary dental lamina. As expected, *RUNX2* was also expressed in the developing alveolar bone (Figures 4J–L). Interestingly, there was evidence of upregulation associated with all three genes in mesenchyme of the cervical loop on the buccal side (Figures 4E,H,K; F,I,L black arrows).

## Discussion

The molecular basis of successional tooth initiation is poorly understood and limited in comparison to what is known about the primary dentition. However, it has been recently suggested that Sox2 may represent a marker of epithelial competence during tooth generation in mammals and reptiles, both for successional tooth formation and serial addition of molars. Although *Sox2* conditional mouse mutants only demonstrate hyperplasia within the developing molar dentition, these findings support the idea that a dormant capacity for tooth renewal does exist within mammals (Juuri et al., 2013).

Additional (or supernumerary) tooth formation is seen in human populations, most commonly occurring as an isolated trait and associated with rudimentary incisor teeth in the anterior maxilla, supplemental supernumeraries, and odontomes (Cobourne and Sharpe, 2010, 2013). However, a number of well-defined syndromic conditions also have additional tooth formation as a feature, including Cleidocranial dysplasia (CCD) [#119600] and the Familial Adenomatous Polyposis [**#**175100] variant Gardner syndrome (Fader et al., 1962). In CCD, multiple supplemental supernumerary teeth are seen in both jaws, particularly affecting the successional dentition (Kreiborg et al., 1999). The RUNX2 transcription factor is therefore a potential regulator of the successional lamina, most-likely associated with tooth germs within the permanent dentition. In the mouse embryo, *Runx2* is expressed in the mesenchymal compartment of the tooth germ and regulated by FGF signaling (Aberg et al., 2004b); however, tooth development arrests at the bud stage in *Runx2* mutant mice (Aberg et al., 2004a; Wang et al., 2005). In the developing Ferret dentition, *Runx2* is expressed in the dental papilla and follicle of the primary tooth germ and in mesenchyme adjacent to the successional lamina, although no expression on the lingual side of the dental lamina was observed (Jussila et al., 2014). Here, we only observed relatively low expression in the mesenchymal compartment of the tooth and no specific association between *RUNX2* expression domains and the successional lamina. This is perhaps surprising; however, we have investigated primary tooth germs at a relatively early stage of development in this study. The supernumerary teeth that are seen in CCD are most commonly associated with the permanent incisor and premolar dentition, rather than the primary teeth. This suggests that *RUNX2* might be more specifically associated with the suppression of successional lamina activity in the permanent tooth germs during human postnatal development. In the mouse, there is some weak evidence that Runx2 might be involved in restricting Shh signaling within the developing primary tooth germs. At least in the maxillary molar teeth, prominent lingual epithelial buds have been described, associated with increased Shh pathway activity in the epithelium (Wang et al., 2005).

Spry2 and Gas1 have both been implicated in the negative regulation of Shh signaling during the development of supernumerary premolar-like teeth in the jaw diastema during development of the mouse dentition (Klein et al., 2006; Ohazama et al., 2009). In the mouse, *Spry2* is thought to achieve this through the modulation of Fgf signaling in the epithelial compartment of the tooth germ, consistent with the observed strong expression that is seen in this region, directly adjacent to the mesenchyme and including the enamel knot. *Spry2* demonstrates much lower levels of expression in the dental mesenchyme of the murine tooth, which contrasts with the human tooth germs examined here, where *SPRY2* transcripts were identified strongly in the mesenchymal component as well as the epithelium, a finding that is similar to that seen in the Ferret tooth (Jussila et al., 2014). Although there was some upregulation of *SPRY2* in the buccal cervical loop, there was no clearly discernible expression in the primary or successional laminas. In contrast, *GAS1* did show definable expression in mesenchyme adjacent to the primary dental lamina, a finding consistent with the developing mouse dentition (unpublished observations) and there was also evidence of increased expression adjacent to the successional lamina of human molar teeth. It has not been shown definitively whether the supernumerary premolar teeth seen in *Gas1* mutant mice are vestigial in nature, but the highly penetrant nature of the extra teeth seen in both jaws of these mice demonstrates the importance of the encoded membrane protein in the regulation of tooth number. Given the expression of *GAS1* during human tooth development described here, and previous findings of a role during human craniofacial development (Ribeiro et al., 2010), it is reasonable to speculate that *GAS1* may represent a candidate gene for supernumerary tooth formation in human populations.

*SPRY2*, *GAS1*, and *RUNX2* were all expressed during the early stages of human tooth development within mesenchymal compartments of the tooth germ. The expression domains of *GAS1* and *RUNX2* were consistent with a role influencing formation of the secondary dentition.

### Conflict of interest statement

The authors declare that the research was conducted in the absence of any commercial or financial relationships that could be construed as a potential conflict of interest.

## References

[B1] Aberg T. Cavender A. Gaikwad J. S. Bronckers A. L. Wang X. Waltimo-Siren J.. (2004a). Phenotypic changes in dentition of Runx2 homozygote-null mutant mice. J. Histochem. Cytochem. 52, 131–139. 10.1177/002215540405200113 14688224

[B2] Aberg T. Wang X. P. Kim J. H. Yamashiro T. Bei M. Rice R.. (2004b). Runx2 mediates FGF signaling from epithelium to mesenchyme during tooth morphogenesis. Dev. Biol. 270, 76–93. 10.1016/j.ydbio.2004.02.012 15136142

[B3] Ahn Y. Sanderson B. W. Klein O. D. Krumlauf R. (2010). Inhibition of Wnt signaling by Wise (Sostdc1) and negative feedback from Shh controls tooth number and patterning. Development 137, 3221–3231. 10.1242/dev.054668 20724449 PMC6512258

[B4] Berkovitz B. K. B. Holland G. R. Moxham B. J. (2009). Oral Anatomy, Embryology and Histology, 4th Edn. Edinburgh: Mosby International Ltd.

[B5] Buchtova M. Handrigan G. R. Tucker A. S. Lozanoff S. Town L. Fu K.. (2008). Initiation and patterning of the snake dentition are dependent on Sonic hedgehog signaling. Dev. Biol. 319, 132–145. 10.1016/j.ydbio.2008.03.004 18456251

[B6] Charles C. Hovorakova M. Ahn Y. Lyons D. B. Marangoni P. Churava S.. (2011). Regulation of tooth number by fine-tuning levels of receptor-tyrosine kinase signaling. Development 138, 4063–4073. 10.1242/dev.069195 21862563 PMC3160100

[B7] Cobourne M. T. Sharpe P. T. (2010). Making up the numbers: the molecular control of mammalian dental formula. Semin. Cell Dev. Biol. 21, 314–324. S1084–S9521. 10.1016/j.semcdb.2010.01.007 20080198

[B8] Cobourne M. T. Sharpe P. T. (2013). Diseases of the tooth: the genetic and molecular basis of inherited anomalies affecting the dentition. Wiley Interdiscip. Rev. Dev. Biol. 2, 183–212. 10.1002/wdev.66 24009033

[B9] Fader M. Kline S. N. Spatz S. S. Zubrow H. J. (1962). Gardner's syndrome (intestinal polyposis, osteomas, sebaceous cysts) and a new dental discovery. Oral Surg. Oral Med. Oral Pathol. 15, 153–172. 10.1016/0030-4220(62)90004-X 13891268

[B10] Handrigan G. R. Richman J. M. (2010a). Autocrine and paracrine Shh signaling are necessary for tooth morphogenesis, but not tooth replacement in snakes and lizards (Squamata). Dev. Biol. 337, 171–186. 10.1016/j.ydbio.2009.10.020 19850027

[B11] Handrigan G. R. Richman J. M. (2010b). A network of Wnt, hedgehog and BMP signaling pathways regulates tooth replacement in snakes. Dev. Biol. 348, 130–141. 10.1016/j.ydbio.2010.09.003 20849841

[B12] Jarvinen E. Tummers M. Thesleff I. (2009a). The role of the dental lamina in mammalian tooth replacement. J. Exp. Zool. B Mol. Dev. Evol. 312B, 281–291. 10.1002/jez.b.21275 19137538

[B13] Jarvinen E. Tummers M. Thesleff I. (2009b). The role of the dental lamina in mammalian tooth replacement. J. Exp. Zool. B Mol. Dev. Evol. 312B, 281–291. 10.1002/jez.b.21275 19137538

[B14] Jernvall J. Thesleff I. (2000). Reiterative signaling and patterning during mammalian tooth morphogenesis. Mech. Dev. 92, 19–29. 10.1016/S0925-4773(99)00322-6 10704885

[B15] Jernvall J. Thesleff I. (2012). Tooth shape formation and tooth renewal: evolving with the same signals. Development 139, 3487–3497. 10.1242/dev.085084 22949612

[B16] Jussila M. Crespo Yanez X. Thesleff I. (2014). Initiation of teeth from the dental lamina in the ferret. Differentiation 87, 32–43. 10.1016/j.diff.2013.11.004 24393477

[B17] Juuri E. Jussila M. Seidel K. Holmes S. Wu P. Richman J.. (2013). Sox2 marks epithelial competence to generate teeth in mammals and reptiles. Development 140, 1424–1432. 10.1242/dev.089599 23462476 PMC3596986

[B18] Klein O. D. Minowada G. Peterkova R. Kangas A. Yu B. D. Lesot H.. (2006). Sprouty genes control diastema tooth development via bidirectional antagonism of epithelial-mesenchymal FGF signaling. Dev. Cell. 11, 181–190. 10.1016/j.devcel.2006.05.014 16890158 PMC2847684

[B19] Komori T. Yagi H. Nomura S. Yamaguchi A. Sasaki K. Deguchi K.. (1997). Targeted disruption of Cbfa1 results in a complete lack of bone formation owing to maturational arrest of osteoblasts. Cell 89, 755–764. 10.1016/S0092-8674(00)80258-5 9182763

[B20] Kreiborg S. Jensen B. L. Larsen P. Schleidt D. T. Darvann T. (1999). Anomalies of craniofacial skeleton and teeth in cleidocranial dysplasia. J. Craniofac. Genet. Dev. Biol. 19, 75–79. 10416150

[B21] Lee B. Thirunavukkarasu K. Zhou L. Pastore L. Baldini A. Hecht J.. (1997). Missense mutations abolishing DNA binding of the osteoblast-specific transcription factor OSF2/CBFA1 in cleidocranial dysplasia. Nat. Genet. 16, 307–310. 10.1038/ng0797-307 9207800

[B22] Lesot H. Hovorakova M. Peterka M. Peterkova R. (2014). Three-dimensional analysis of molar development in the mouse from the cap to bell stage. Aust. Dent. J. 59(Suppl. 1), 81–100. 10.1111/adj.12132 24495111

[B23] Mundlos S. Otto F. Mundlos C. Mulliken J. B. Aylsworth A. S. Albright S.. (1997). Mutations involving the transcription factor CBFA1 cause cleidocranial dysplasia. Cell 89, 773–779. 10.1016/S0092-8674(00)80260-3 9182765

[B24] Ohazama A. Haycraft C. J. Seppala M. Blackburn J. Ghafoor S. Cobourne M.. (2009). Primary cilia regulate Shh activity in the control of molar tooth number. Development 136, 897–903. 10.1242/dev.027979 19211681 PMC2727556

[B25] Peterkova R. Hovorakova M. Peterka M. Lesot H. (2014). Three-dimensional analysis of the early development of the dentition. Aust. Dent. J. 59(Suppl. 1), 55–80. 10.1111/adj.12130 24495023 PMC4199315

[B26] Ribeiro L. A. Quiezi R. G. Nascimento A. Bertolacini C. P. Richieri-Costa A. (2010). Holoprosencephaly and holoprosencephaly-like phenotype and GAS1 DNA sequence changes: report of four Brazilian patients. Am. J. Med. Genet. A 152A, 1688–1694. 10.1002/ajmg.a.33466 20583177

[B27] Seppala M. Depew M. J. Martinelli D. C. Fan C. M. Sharpe P. T. Cobourne M. T. (2007). Gas1 is a modifier for holoprosencephaly and genetically interacts with sonic hedgehog. J. Clin. Invest. 117, 1575–1584. 10.1172/JCI32032 17525797 PMC1868789

[B28] Seppala M. Xavier G. M. Fan C. M. Cobourne M. T. (2014). Boc modifies the spectrum of holoprosencephaly in the absence of Gas1 function. Biol. Open. 3, 728–740. 10.1242/bio.20147989 25063195 PMC4133726

[B29] Ten Cate A. R. (2014). Oral Anatomy: Development, Structure and Function. 8th Edn, St Louis, MO: Mosby-Year Book Inc.

[B30] Tucker A. S. Fraser G. J. (2014). Evolution and developmental diversity of tooth regeneration. Semin. Cell Dev. Biol. 25–26, 71–80. 10.1016/j.semcdb.2013.12.013 24406627

[B31] Tucker A. S. Sharpe P. (2004). The cutting edge of mammalian development; how the embryo makes teeth. Nat. Rev. Genet. 5, 499–508. 10.1038/nrg1380 15211352

[B32] Wang X. P. Aberg T. James M. J. Levanon D. Groner Y. Thesleff I. (2005). Runx2 (Cbfa1) inhibits Shh signaling in the lower but not upper molars of mouse embryos and prevents the budding of putative successional teeth. J. Dent. Res. 84, 138–143. 10.1177/154405910508400206 15668330

[B33] Wilkinson D. G. (1992). In situ Hybridisation: A Practical Approach. Oxford, UK: IRL Press.

[B34] Yamanaka A. Yasui K. Sonomura T. Iwai H. Uemura M. (2010). Development of deciduous and permanent dentitions in the upper jaw of the house shrew (Suncus murinus). Arch. Oral Biol. 55, 279–287. 10.1016/j.archoralbio.2010.02.006 20303065

