# Corrigendum: Expression analysis of candidate genes regulating successional tooth formation in the human embryo

**DOI:** 10.3389/fphys.2015.00041

**Published:** 2015-02-12

**Authors:** Ryan Olley

**Affiliations:** Department of Prosthodontics, Kings College LondonLondon, UK

**Keywords:** human tooth development, three-dimensional reconstruction, SPROUTY2, GAS1, RUNX2, primary dental lamina, successional dental lamina, gene expression

The author Ryan Olley should appear as Olley RC on the published article “Expression analysis of candidate genes regulating successional tooth formation in the human embryo.”

The original article was updated.

## Conflict of interest statement

The author declares that the research was conducted in the absence of any commercial or financial relationships that could be construed as a potential conflict of interest.

